# New Insights in Cheese Yield Capacity of the Milk of Italian Brown and Italian Friesian Cattle in the Production of High-Moisture Mozzarella

**DOI:** 10.17113/ftb.58.01.20.6386

**Published:** 2020-03

**Authors:** Piero Franceschi, Massimo Malacarne, Michele Faccia, Attilio Rossoni, Enrico Santus, Paolo Formaggioni, Andrea Summer

**Affiliations:** 1 Department of Veterinary Science, University of Parma, Strada del Taglio 10, 43126 Parma, Italy; 2 Department of Soil, Plant and Food Sciences (Di.S.S.P.A.), University of Bari, Via G. Amendola 165/A, 70126 Bari, Italy; 3 ANARB - Italian Brown Cattle Breeders Association, Loc. Ferlina 204, 37012 Bussolengo (VR), Italy

**Keywords:** mozzarella cheese, cheese yield, cheese making losses, Italian Brown cattle, κ-casein B

## Abstract

The aim of the present study is to investigate the effect of κ-casein B content in milk on the yield of high-moisture mozzarella cheese. The study was carried out by monitoring the production of eight mozzarella cheese batches at four cheese making factories. At each factory, two cheese making trials were performed in parallel: one using bulk milk from Italian Brown cattle and the other using bulk milk from Italian Friesian cattle. The average κ-casein B content was 0.04 g per 100 g in the Italian Friesian cows’ milk, whereas it was four time higher in the Italian Brown cows’ milk, reaching values of 0.16 g per 100 g. Both the κ-casein content and κ-casein B to casein ratio were positively correlated with actual cheese yield. Both parameters showed correlation coefficient values over 0.9, higher than for any other protein fraction. The influence of the level of κ-casein on the increase of the yield is probably due to smaller and more homogeneous micelles, with more efficient rennet coagulation. Consequently, milk with higher κ-casein B content produces a more elastic curd that withstands better the technological treatments and limits losses during curd mincing and stretching. In conclusion, the Italian Brown cows’ milk used, characterized by higher κ-casein content than the Italian Friesian’s one, allowed a yield increase of about 2.65%, which is a very relevant result for both farms and cheese making factories.

## INTRODUCTION

The profitability of a cheese-making factory industry is based on the cheese yield capacity of the processed milk, which, in turn, depends on many factors. Since cheese is formed by a paracasein network that incorporates fat globules and milk whey, the most important and studied factors that affect cheese yield are milk casein content, casein composition and casein polymorphisms. Regarding casein polymorphism, the two widely diffuse genetic variants of κ-casein (κ-CN) are designated as A and B (*1*). κ-Casein B is associated with higher casein content in milk (*2*), and higher percentage of κ-casein in total casein than κ-casein A (*3*). These characteristics result in smaller micelle size (*4*) that, in turn, leads to improved curd properties (*2*) and higher cheese yield, as observed in the cheesemaking of Cheddar (*4*, *5*), Parmigiano Reggiano (*6*) and partially skimmed Mozzarella (*7*).

The most relevant Italian types of cheese obtained from cow’s milk are Grana Padano, Parmigiano Reggiano and high-moisture mozzarella produced with full cream milk. This last cheese is very different from the low-moisture type. The yield of high-moisture mozzarella ranges from 12 to 15% and these variations strongly influence the viability and profitability of the cheese factories.

In Italy, as well as in the production of high-moisture mozzarella cheese, the two most important breeds for milk production are Italian Brown and Italian Friesian. On average, milk from Italian Brown is characterised by higher fat, higher protein and higher casein contents (*8*, *9*), as well as higher percentage of κ-casein B, better rennet coagulation properties (*9*) and, finally, higher mineral (mainly calcium, phosphorus and magnesium) content (*8*).

To our knowledge, the effect of κ-casein B content on cheese making yield of high-moisture mozzarella has never been investigated. Although Walsh *et al.* (*7*) have already conducted a study on low-moisture mozzarella cheese, that study was conducted under laboratory and small-scale conditions, and made comparison only between homozygote variants of κ-casein (κ-CN AA *vs* κ-CN BB). Furthermore, that study reported the difference among the cheese-making yield capacity of milk containing different variants of κ-casein but did not investigate adequately the mechanism that causes this difference in yield.

Considering the relevance of this subject for the cheese-making factories, the aim of the present study is to investigate the factors affecting high-moisture mozzarella cheese yield. We put special emphasis on the roles of milk κ-casein B, in particular the effect of milk κ-casein B content on milk composition and its repercussion on cheese yield and on cheesemaking losses in high-moisture mozzarella manufacturing.

## MATERIALS AND METHODS

### Experimental design, sampling procedure and classification of cheese batches

The raw milk used for cheese making was collected from Italian Friesian and Italian Brown pure breed herds reared in Apulia (Italy). Seven herds participated in the study (3 of Italian Friesian and 4 of Italian Brown), all reared under the local semi-free farming technique. In this technique, the cattle are free to move out of the stable and graze on natural or cultivated pastures (depending on availability) for 3-4 h a day throughout the year (weather permitting). The size of the herds ranged from 50 to 150 lactating cows, with average milk production per cow of about 35 L/day (Italian Friesian) and about 24 L/day (Italian Brown). The study was carried out following the production of eight mozzarella cheese batches in four cheese factories located in Apulia Region (Italy): three of them are at Gioia del Colle (Bari, Italy) and one at Putignano (Bari, Italy). In each, two cheese-making trials were followed in parallel: one using bulk tank milk obtained from Italian Brown cattle herds and the other using bulk tank milk obtained from Italian Friesian cattle herds.

Bulk tank milk contained approx. 500-1000 kg of milk representative of morning milking of each herd involved in the study. Each Italian Friesian herd supplied about 800 kg milk for the experimentation, whereas each Italian Brown herd supplied about 600 kg milk. Each batch of milk was carefully weighed before filling the tank, and a sample was taken before adding the natural whey starter. At the end of the cheese making process, a sample of residual milk whey was collected from the tank. The cheese obtained from each trial was weighed after cooling, and samples were taken for analysis.

### Analytical methods

The following parameters of each bulk tank milk sample were determined: total nitrogen, non-casein nitrogen and non-protein nitrogen (NPN) in milk, acid whey at pH=4.6 and trichloroacetic acid (TCA)-filtered whey (obtained by adding TCA at the final concentration of 120 g/L in milk; Carlo Erba Reagents, Milan, Italy), respectively, by Kjeldahl method (*10*) using a DK6 digestor and an UDK126A distiller (VELP Scientifica, Usmate, Italy). From these data, crude protein, casein, whey protein, NPN×6.38 and true protein content was calculated as described by Malacarne *et al.* (*8*). Similarly, crude protein, whey protein and casein were determined also in milk whey, using the same method. κ-Casein B content was determined in bulk tank milk with ELISA method (Astori Tecnica s.n.c., Poncarale (BS), Italy), using specific antibody according to Summer *et al.* (*1*), and κ-casein B to casein ratio was calculated. Fat content of bulk tank milk was determined by using mid-infrared spectrometer MilkoScan FT 6000 (Foss Electric, Hillerød, Denmark) and fat content of milk whey by volumetric Gerber method (*11*). Dry matter was obtained after oven drying at 102 °C and ash after muffle calcination at 530 °C of both bulk tank milk and whey. Moreover, each milk sample, previously skimmed, was also ultrafiltered in Amicon 8200 ultrafiltration cells (Merck Millipore Corporation, Darmstadt, Germany). The ultrafiltration was performed as described by Malacarne *et al.* (*12*) with a Millipore membrane with 30 kDa cut-off using a N_2_ flow at 517 kPa (polyethersulfone ultrafiltration membranes, Merck Millipore Corporation). Total Ca and Mg in milk and soluble Ca and Mg in ultrafiltration permeate were determined in hydrochloric ash solution by atomic absorption spectrometry (1100 B; Perkin-Elmer, Waltham, MA, USA) (*13*). Total P, soluble P and total acid-soluble P were assessed in milk, in ultrafiltration permeate and in milk after treatment with the TCA (120 g/L), respectively, with the colorimetric method proposed by Allen (*14*). Distribution of Ca, P and Mg fractions was calculated according to Malacarne *et al.* (*15*).

Somatic cells and total bacterial count of each bulk tank milk sample were determined by fluoro-opto-electronic method with Fossomatic FC (Foss Electric) and by flow cytometry method with BactoScan FC (Foss Electric), respectively. Curd fines of each milk whey sample were measured as described by Franceschi *et al*. (*16*). In this method, 250 mL of whey were centrifuged at 2000×*g* for 30 min. The pellet was resuspended in distilled water and filtered through a Whatman no. 40 filter paper. The filter was dried at 102 °C for 2 h and weighed.

Moreover, bulk tank milk before the natural whey starter addition and the cheese obtained from each cheese-making process were weighted. Fat, crude protein and moisture of each mozzarella cheese sample were determined by volumetric Gerber method (*11*), Kjeldahl method (*10*) and after drying at 102 °C, respectively.

The actual cheese yield (kg of cheese per 100 kg of milk) was calculated as follows:

*Y* =*m*_c_·100/*m*_m_ /1/

where *Y* is actual cheese yield of milk, *m*_c_ is cheese mass (in kg), and *m*_m_ is milk mass (in kg).

The adjusted dry cheese yield and the adjusted yield at 60% of moisture were calculated according to the following formula:

*Y*_ad_=*Y*·[100-*w*_r_(moisture)/(100-*w*_d_(moisture)] /2/

where *Y*_ad_ is adjusted yield (dry cheese or at 60% moisture), *w*_r_(moisture) is real moisture content, and *w*_d_(moisture) is desirable moisture content (0% for dry cheese or 60%).

### Statistical analysis

The batches of cheese were divided into two classes according to the breed: Italian Friesian and Italian Brown. The significance of the differences between classes was tested by analysis of variance, using the general linear model procedure of SPSS v. (*17*), according to the following univariate model:

Y_ijk_=µ+C_i_+T_j_+ε_ijk_ /3/

where Y_ijk_ is dependent variable, µ is overall mean, C_i_ is the effect of breed class (i=1, 2), T_j_ is the effect of trial (j=1,….4); ε_ijk_ is residual error. The significance of the differences was tested by means of least significant differences method. Data were also processed by the Pearson product-moment correlation coefficient to measure the degree of linear relationship between parameters characteristic for milk and cheese yield.

## RESULTS AND DISCUSSION

### Milk characteristics and cheese yield

The mean values of the bulk tank milk parameters and their correlation coefficients with actual cheese yield are shown in Table 1. Among the protein fractions, the values of crude protein, casein and true protein were positively correlated with actual cheese yield with coefficient values over 0.7. Also, fat content positively correlated with actual cheese yield, with a coefficient value over 0.7. This evidence was observed in different types of cheese production, *i.e.* in Saint-Nectaire cheese (*18*), Grana Padano cheese (*19*) and Parmigiano Reggiano cheese (*20*).

**Table 1:** Characteristics of bulk tank milk (*N*=8) and their Pearson correlation coefficient with the actual cheese yield (ACY). Only significant correlations (p<0.05) are reported

Parameter	Descriptive statistics						Pearson correlation coefficient	
	Mean		S.D.	Minimum	Maximum		ACY	p-value
*w*(dry matter)/(g/100 g)	13.16		0.29	12.65	13.52		0.730	*
*w*(fat)/(g/100 g)	3.75		0.09	3.60	3.85		0.758	*
*w*(crude protein)/(g/100 g)	3.47		0.19	3,30	3.77		0.794	*
*w*(whey protein)/(g/100 g)	0.81		0.08	0.66	0.89			NS
*w*(casein)/(g/100 g)	2.65		0.17	2.47	2.89		0.761	*
*w*(NPN×6.38)/(g/100 g)	0.17		0.05	0.11	0.26			NS
*w*(true protein)/(g/100 g)	3.29		0.17	3.10	3.59		0.825	*
*w*(κ-casein B)/(g/100 g)	0.10		0.07	0.04	0.17		0.933	***
(*m*(κ-casein B)/*m*(casein))/%	3.65		2.29	1.40	6.16		0.920	***
*w*(ash)/(g/100 g)	0.73		0.01	0.72	0.75		0.718	*
*w*(total Ca)/(mg/100 g)	114.31		3.93	110.03	121.87		0.672	*
*w*(soluble Ca)/(mg/100 g)	23.93		2.14	21.62	26,71			NS
*w*(colloidal Ca)/(mg/100 g)	90.38		3.01	87.03	96.46			NS
*w*(total P)/(mg/100 g)	93.30		2.01	90.60	95.90		0.774	*
*w*(soluble P)/(mg/100 g)	38.74		5.90	30.62	47.69		-0.828	*
*w*(colloidal P)/(mg/100 g)	52.28		7.17	42.82	61.15		0.892	**
*w*(colloidal inorganic P)/(mg/100 g)	31.41		6.64	21.52	39.87		0.792	*
*w*(casein P)/(mg/100 g)	20.86		2.02	18.31	24.48			NS
*w*(total Mg)/(mg/100 g)	11.01		0.36	10.49	11.56			NS
*w*(soluble Mg)/(mg/100 g)	8.31		0.26	7.85	8.68			NS
*w*(colloidal Mg)/(mg/100 g)	2.70		0.16	2.45	2.89			NS
*N*(somatic cell)/(10^3^ cell/mL)^a^	289		47	253	363		-0.753	*
*N*(total bacteria)/(10^3^ CFU/mL)^a^	28		8	25	36			NS

NPN=non-protein nitrogen; NS=not significant (p>0.05), *p≤0.05, **p≤0.01, ***p≤0.001

^a^Pearson correlation coefficient is calculated between the actual cheese yield and logarithmic values of somatic cell count and total bacterial count

The κ-casein B content and κ-casein B to casein ratio were on average 0.10 g/ 100 g and 3.65%, respectively, and both positively correlated with actual cheese yield. Both parameters showed coefficient values over 0.9, higher than for any other protein fraction. The strong link between κ-casein B content and the actual cheese yield is consistent with that shown by Walsh *et al.* (*7*) for the low-moisture mozzarella cheese produced with partially skimmed milk.

Ash value was positively correlated with the actual cheese yield. This relation is mainly due to calcium and phosphorus contents, both positively correlated with actual cheese yield. The average values for ash, calcium and phosphorus are in accordance with those reported by Summer *et al.* (*21*) in a research on milk used for manufacturing Parmigiano Reggiano cheese, which showed a significant Pearson correlation coefficient between phosphorus content and actual cheese yield (r=0.036; p≤0.05). Colloidal phosphorus and inorganic colloidal phosphorus contents were positively correlated with actual cheese yield, while on the contrary, soluble phosphorus content was negatively correlated with actual cheese yield. The first observation mainly depends on the relationship existing between casein and actual cheese yield, especially in milk with high casein content since approximately half of the phosphorus contained in milk is associated with casein (*15*, *22*). On the other hand, it is difficult to explain the second observation, which was partially in discordance with those reported by other authors. For example, Malacarne *et al.* (*15*) showed that milk with better milk coagulation properties and consequently, in general, with higher cheese yield capacity (*19*) had higher soluble phosphorus content. However, it must be noted that highly mineralised micelles, with a high content of colloidal phosphorus, had already been described in the past by Petrera *et al.* (*23*) and Summer *et al.* (*24*) for milk of Modenese cow breed, where the soluble phosphorus fraction was also lower than normal. These types of milk are characterised by anomalous milk coagulation properties and produce curd with low consistence and friability. This curd is unsuitable for the processing of the technological steps of the transformation of milk into dairy products (*24*), which can result in cheese yield reduction. Finally, a negative correlation was shown between somatic cell content and actual cheese yield. This is widely attested in literature in many types of cheese production: *i.e.* in Cheddar (*25*), cottage (*26*), Grana Padano (*19*) and Parmigiano Reggiano (*21*). This phenomenon is mainly caused by the negative modification of chemical composition and physicochemical properties of milk with high somatic cell content (*27*). In particular, in milk with high somatic cell content, there is a decrease of important constituents like casein, which is the primary matter of the cheese (*28*). In milk with a high somatic cell content, there is also a decrease of main milk mineral contents like phosphorus, which causes the worsening of milk coagulation properties, as reported by Summer *et al*. (*29*) in a research comparing milk produced by two quarters (rear or front) of the same cow: one quarter with milk somatic cell count lower and the other with higher than 400 000 cell/mL. Finally, in the present research, somatic cells ranged from 253 000 to 363 000 cell/mL; these data confirm the observation reported by Summer *et al.* (*21*) for Parmigiano Reggiano cheese making, also for high-moisture mozzarella, that a reduction of milk cheese yield capacity occurs starting from 300 000 cell/mL.

### Difference in the milk characteristics between the two breeds

The comparison between the least square mean values of characteristics of the milk of Italian Brown and Italian Friesian breeds are shown in Table 2. Dry matter, crude protein, casein, true protein, ash and total Ca were higher in the milk of Italian Brown than of Italian Friesian breed, with p≤0.05. Moreover, colloidal, colloidal inorganic and casein phosphorus were higher in the former, also with p≤0.05 as well as total phosphorus, with p≤0.01. On the contrary, the total bacterial count was lower in the milk of Italian Brown than of the Italian Friesian one. However, although the difference among the mean values was significant with p≤0.01, the amount of the difference is too small to affect the milk quality. Both mean values of total bacterial count were well below the threshold value of 100 000 FCU/mL reported by Summer *et al.* (*30*) for non-law-complying milk. Finally, κ-casein B content and κ-casein B to casein ratio were higher in the milk of Italian Brown than of Italian Friesian breed with p≤0.001. However, it must be noted that the values of κ-casein B in the milk of Italian Brown were always very high. The κ-casein B content of milk was on average 0.04 g per 100 g in that of Italian Friesian, while in that of Italian Brown this parameter was four times higher, reaching values of 0.16 g per 100 g.

**Table 2:** Comparison between the least square mean values of parameters of Italian Friesian cows’ milk (IF; *N*=4) and Italian Brown cows’ milk (IB; *N*=4)

Parameter	IF		IB		SE		p-value
*w*(dry matter)/(g/100 g)	12.95		13.37		0.10		*
*w*(fat)/(g/100 g)	3.68		3.83		0.03		**
*w*(crude protein)/(g/100 g)	3.33		3.60		0.07		*
*w*(casein)/(g/100 g)	2.53		2.77		0.06		*
*w*(κ-casein B)/(g/100 g)	0.04		0.16		0.01		***
(*m*(κ-casein B)/*m*(casein))/%	1.52		5.79		0.11		***
*w*(NPN×6.38)/(g/100 g)	0.17		0.18		0.02		NS
*w*(true protein)/(g/100 g)	3.16		3.43		0.05		*
*w*(ash)/(g/100 g)	0.72		0.74		0.01		*
*w*(total Ca)/(mg/100 g)	111.38		117.24		1.28		*
*w*(soluble Ca)/(mg/100 g)	22.60		25.25		1.14		NS
*w*(colloidal Ca)/(mg/100 g)	88.78		91.99		0.65		*
*w*(total P)/(mg/100 g)	91.59		95.01		0.46		**
*w*(soluble P)/(mg/100 g)	42.62		34.85		3.02		NS
*w*(colloidal P)/(mg/100 g)	46.73		57.82		3.03		*
*w*(colloidal inorganic P)/(mg/100 g)	27.33		35.49		3.67		*
*w*(casein P)/(mg/100 g)	19.40		22.33		0.73		*
*w*(total Mg)/(mg/100 g)	10.84		11.16		0.17		NS
*w*(soluble Mg)/(mg/100 g)	8.22		8.39		0.12		NS
*w*(colloidal Mg)/(mg/100 g)	2.63		2.77		0.05		NS
*N*(somatic cell count)/(10^3^ cell/mL)^a^	318		258		18		NS
*N*(total bacterial count)/(10^3^ CFU/mL)^a^	22		34		2		**

SE=standard error, NPN=non-protein nitrogen; NS=not significant (p>0.05), *p≤0.05, **p≤0.01, ***p≤0.001

^a^Significance of differences for somatic cell count and total bacterial count is calculated on logarithmic values

According to data reported by Marziali and Ng-Kwai-Hang (*5*) and Walsh *et al.* (*4*, *7*), who compared κ-CN AA with κ-CN BB milk, and the results of Comin *et al.* (*2*), who compared milk samples with different levels of κ-casein B, higher content of κ-casein B in the milk of Italian Brown cattle resulted in higher casein and protein contents. Because the casein micelles consist of caseins (α_S1_, α_S2_, β- and κ-casein) and calcium phosphate, the greater content of casein results in a higher content of colloidal calcium and colloidal phosphorus, both as inorganic phosphorus and casein phosphorus. This, in turn, leads to a higher content of total calcium and total phosphorus and consequently higher ash contents in the milk of Italian Brown than of Italian Friesian cattle. The higher crude protein and casein content of the milk of Italian Brown than of the Italian Friesian cattle was already reported by De Marchi *et al.* (*9*), who in a research conducted on the milk from 506 single-breed herd reported a higher protein content in the milk of Italian Brown than in that of Italian Friesian one (3.48 *vs* 3.19 g per 100 g). Similarly, Malacarne *et al.* (*8*) showed higher protein (3.49 *vs* 3.07 g per 100 g) and casein (2.71 *vs* 2.37 g per 100 g) contents, as well as higher total (120.76 *vs* 113.19 mg per 100 g) and colloidal (83.32 *vs* 74.25 mg per 100 g) calcium contents and higher total (97.32 *vs* 88.04 mg per 100 g) and colloidal (50.00 *vs* 44.40 mg per 100 g) phosphorus contents in the milk of Italian Brown.

### Difference between the milk cheese yield of different breeds and the cheese making losses

Least square mean values of the cheese yield and characteristics of mozzarella cheese obtained from the milk of Italian Friesian and Italian Brown breed are shown in Table 3 and the least square mean values of cheese making losses of these milk samples are shown in Table 4.

**Table 3:** Least square mean values of cheese yield and parameters of mozzarella cheese obtained from the milk of Italian Friesian (IF; *N*=4) and Italian Brown breeds (IB; *N*=4)

Parameter	IF		IB		SE		p-value
Cheese yield:	*Y*/(kg/100 kg)						
Actual cheese	12.86		15.51		0.32		***
Dry cheese	5.61		7.07		0.31		*
Adjusted cheese yield^a^	14.04		17.68		0.78		*
Cheese characteristics:	*w*/(g/100 kg)						
Moisture	56.38		54.39		2.07		NS
Crude protein	20.69		22.16		0.99		NS
Fat	20.75		20.99		1.80		NS

SE=standard error, NS=not significant (p>0.05), *p≤0.05, ***p≤0.001

^a^Yield adjusted at 60% moisture content

**Table 4:** Least square mean values of the cheese making losses and curd fines of the milk of Italian Friesian (IF; *N*=4) and Italian Brown breeds (IB; *N*=4)

Parameter	IF		IB		SE			p-value
	*w*(parameter)_loss_/%							
Dry matter	55.99		51.39		0.91			**
Protein	28.05		23.27		1.11			*
Casein	2.61		1.75		0.53			*
Fat	14.95		15.04		0.68			NS
Ash	77.45		74.89		0.41			**
Phosphorus	54.85		53.89		0.84			NS
Calcium	52.98		50.30		1.97			NS
Magnesium	76.71		75.26		1.79			NS
	*w*/(mg/kg)							
Curd fines	554		338		89			*

SE=standard error, NS=not significant (p>0.05), *p≤0.05, **p≤0.01

Actual cheese yield was higher from the milk of Italian Brown breed than of the milk from Italian Friesian breed with p≤0.001. Also, dry yield and yield adjusted at 60% moisture content values were higher in the former with p≤0.05. Moreover, protein and casein losses, and curd fines content were different in the milk obtained from the both Italian breeds with p≤0.05. However, in this case all the above parameters were higher in cheese making from the milk of Italian Friesian than Italian Brown one. Finally, dry matter losses and ash losses were higher in the cheese-making process with the milk of Italian Friesian than of Italian Brown breed, both with p≤0.01.

The actual high-moisture mozzarella cheese yield was higher from the milk of Italian Brown breed than from that of Italian Friesian one. This is in agreement with the results reported in literature (*5*, *7*), indicating a higher cheese yield capacity of κ-CN BB milk than of κ-CN AA or AB milk in the transformation into low-moisture mozzarella cheese. This fact confirms that this difference also exists in field conditions, as well as in experimental conditions used by Marziali and Ng-Kwai-Hang (*5*) and Walsh *et al.* (*7*). Moreover, this difference is not related to a different content of water that remains in the cheese paste, because apart from actual cheese yield, the dry yield and 60% moisture adjusted yield were higher in the milk of Italian Brown than of Italian Friesian. This observation is confirmed by the similar moisture content that characterises the high-moisture mozzarella produced with both types of milk.

It must be noted that κ-casein B content influences the actual cheese yield with an effect that goes beyond the effect of the increase of milk casein content, as reported in the literature by Ng-Kwai-Hang *et al.* (*31*). This added effect of κ-casein B content on milk cheese yield can be explained with the lower cheese making losses of the milk from Italian Brown than from the Italian Friesian breed.

Among the cheese making losses ((*m*(cheese whey)/*m*(milk))·100), in fact, protein and casein losses were lower in Italian Brown cows’ milk than in Italian Friesian ones’; moreover, the losses of dry matter and ash were also lower in the former. The lower losses of protein compounds of Italian Brown cows’ milk can be explained by the more favourable milk coagulation properties that in general characterise this milk, as reported in the literature by many authors (*8*, *9*). The κ-casein content affects the micelle size and consequently the micelle dispersion. In particular, the increase of the level of κ-casein can lead to smaller and homogeneous micelles that will diffuse easily in the milk (*4*). Small and homogeneous micelles are better for the reaction of casein with rennet enzymes (*5*). Consequently, milk with higher content of κ-casein produces a more elastic and higher quality curd, which can withstand better the technological treatments, with lower protein and casein losses. The better dispersion of casein micelles and the better milk coagulation and curd properties can also explain the lower quantity of fat and curd fines that remain in the milk whey of Italian Brown cows’ milk than of Italian Friesian ones’. Curd fines are very small particles of cheese that are too small to be recovered and for this reason remain in the milk whey. The milk obtained from Italian Brown breed had lower average content of curd fines (38.99%) than the milk from Italian Friesian breed.

## CONCLUSIONS

It can be concluded that the characteristics of the milk of Italian Brown breed used in the present study are more favourable for producing high-moisture mozzarella than that of the Italian Friesian breed. In fact, it allowed a yield increase of about 2.65%, which is a very relevant result for the dairy farms and cheese factories. This difference appears to be too high to be explained only by the higher casein content of milk with higher κ-casein content. In our opinion, the different κ-casein polymorphisms that characterize the milk of the two breeds must also be taken into account, and should be considered as an additional cause of yield increase. The improved milk coagulation ability, but also the properly structured coagulum, are probably at the basis of the observed yield increase. The availability of a well-structured coagulum is pivotal for producing a suitable curd during high-moisture mozzarella production. In fact, the stretching of the curd phase always gives rise to some losses (mainly fat) into the stretching water. Such losses can be minimized if the curd is well composed from the structural and mechanical points of view. This consideration is confirmed by the lower cheese making losses that characterise the milk of Italian Brown breed. For this reason, it would be interesting to invest in the genetic identification and selection of animals to increase the κ-casein B milk content. Finally, additional investigation is needed to deepen the knowledge about the status of calcium and phosphorous salts in the milk of different breeds, and their distribution between soluble and colloidal phase.
